# How effective is tetracaine 4% gel, before a peripherally inserted central catheter, in reducing procedural pain in infants: a randomized double-blind placebo controlled trial [ISRCTN75884221]

**DOI:** 10.1186/1741-7015-4-11

**Published:** 2006-05-03

**Authors:** Brigitte Lemyre, Rebecca Sherlock, Debora Hogan, Isabelle Gaboury, Colline Blanchard, David Moher

**Affiliations:** 1 Department of Pediatrics, Children's Hospital of Eastern Ontario, 401, Smyth Road, Ottawa, Ontario, Canada; 2 Department of Pediatrics, BC Children's and Women's Health Center, 4480, Oak Street, Vancouver, BC, Canada; 3 Department of Nursing, Hospital of Eastern Ontario, 401, Smyth Road, Ottawa, Ontario, Canada; 4 Chalmers Research Group, Children's Hospital of Eastern Ontario Research Institute, 401, Smyth Road, Ottawa, Ontario, Canada; 5 Department of Pharmacy, Children's Hospital of Eastern Ontario, 401, Smyth Road, Ottawa, Ontario, Canada; 6 Department of Epidemiology & Community Medicine, Faculty of Medicine, University of Ottawa, Canada

## Abstract

**Background:**

Procedural pain relief is sub-optimal in infants, especially small and vulnerable ones. Tetracaine gel 4% (Ametop^®^, Smith-Nephew) provides pain relief in children and larger infants, but its efficacy in smaller infants and for peripherally inserted central catheters (PICC) remains uncertain. The objective of this trial was to assess the safety and efficacy of tetracaine gel on the pain response of very low birth weight (VLBW) infants during insertion of a PICC.

**Methods:**

Medically stable infants greater than or equal to 24 weeks gestation, requiring a non-urgent PICC, were included. Following randomization and double blinding, 1.1 g of tetracaine or placebo was applied to the skin for 30 minutes. The PICC was inserted according to a standard protocol. Pain was assessed using the Premature Infant Pain Profile (PIPP). A 3-point change in the pain score was considered clinically significant, leading to a sample size of 54 infants, with 90% statistical power. Local skin reactions and immediate adverse cardiorespiratory events were noted. The primary outcome, PIPP score at 1 minute, was analysed using an independent Student's t-test.

**Results:**

Fifty-four infants were included, 27 +/- 2 weeks gestation, 916 +/- 292 grams and 6.5 +/- 3.2 days of age. Baseline characteristics were similar between groups. The mean PIPP score in the first minute was 10.88 in the treatment group as compared to 11.74 in the placebo group (difference 0.86, 95% CI -1.86, 3.58). Median duration of crying in non-intubated infants was 181 seconds in the tetracaine group compared to 68 seconds in the placebo group (difference -78, 95% CI -539, 117). Local skin erythema was observed transiently in 4 infants (3 in the treatment and 1 in the placebo group). No serious harms were observed.

**Conclusion:**

Tetracaine 4% when applied for 30 minutes was not beneficial in decreasing procedural pain associated with a PICC in very small infants.

## Background

Infants admitted to neonatal intensive care units (NICUs) undergo multiple painful procedures throughout their stay. Those born between 27 and 31 weeks gestation undergo on average 134 painful procedures within the first two weeks of life, ranging from 2 to 8 per day [1,2]. A recent prospective observational study reported a mean of 14.4 painful procedures a day for infants of 25 to 42 weeks gestation [3]. Pain remains under-treated in infants, particularly those who are preterm. There is a growing body of evidence suggesting that multiple painful and stressful events experienced by infants born preterm induce acute physiological changes and permanent structural and functional central nervous system changes [4]. This may lead to several long-term sequelae, including chronic pain and altered neurobehavioral responses to subsequent pain [4]. Our standard of care at the time this protocol was written was to use non-pharmacological interventions, including swaddling and non-nutritive sucking for painful procedures such as venipuncture, heel pricks and placement of peripherally inserted central catheters (PICC). More recently, experts have recommended sucrose and/or local or systemic analgesia before such procedures [5]. Side effects of systemic analgesics limit their use on a regular basis [6].

EMLA (Lidocaine-Prilocaine 5% cream), a widely used anesthetic cream, has a relatively long onset of action (60 minutes), causes local vasoconstriction and is associated with a risk of methemoglobinemia. These factors limit its usefulness in newborns. Tetracaine gel (4% w/w tetracaine in an aqueous gel, Amethocaine 4% or Ametop, Smith-Nephew Inc, St-Laurent, Quebec), a topical anesthetic developed in the early 1990s, may be a promising alternative. The onset of action is 30 to 45 minutes and there is no risk of methemoglobinemia. In addition, it causes local vasodilatation, which may be an advantage when it is used prior to central venous catheter placement.

The localized anesthetic action of tetracaine has been demonstrated after 30 minutes in infants [7]. The evidence supporting the clinical efficacy of tetracaine is equivocal. Two randomized controlled trials (RCTs) reported a reduction in pain scores [8,9] and cry duration [8] during venipuncture and venous cannulation. However, no benefit could be demonstrated for heel pricks in a similar population in two other RCTs [10,11]. A recent randomized trial by Ballantyne et al. [12] did not demonstrate a benefit of tetracaine for PICC insertions in premature infants. This trial included infants 27 weeks or greater.

The evidence is sparse with respect to the efficacy and safety of tetracaine in preterm infants. For PICC insertions specifically, the only trial published is the one from Ballantyne et al., who enrolled 49 infants. There is virtually no evidence about tetracaine for PICC insertion in infants less than 28 weeks, who represent a great proportion of those requiring a PICC. These small infants are subjected to the highest numbers of painful procedures and have the least energy reserves to mount stress reactions. Preterm infants are at the highest risk of suffering long-term consequences of repeated painful experiences [4]. The objective of this study was to evaluate whether the application of tetracaine 4% before inserting a PICC in newborn infants would safely and significantly decrease procedural pain as compared to placebo.

## Methods

### Participants

Infants admitted to the NICU of The Ottawa Hospital, General Campus, Ottawa, Canada were eligible for this study if they required a PICC. The study site is a 25 bed level 2 and 3 NICU at a metropolitan university-affiliated hospital. Infants were enrolled if they met the following inclusion criteria: (a) born at ≥ 24 weeks gestation, (b) skin considered in good condition (no burns or rash), (c) if < 27 weeks gestation, at least 48 h of life and (d) considered stable by the treating neonatologist.

Infants were excluded if they met any of the following criteria: (a) skin considered immature (insensible water losses requiring more fluids than usual for gestation), (b) suspected or proven significant central nervous system anomaly, (c) receiving opioids or sedatives at time of PICC insertion or in the previous 12 h or receiving muscle relaxants, (d) facial anomalies (cleft lip, Moebius syndrome) preventing typical facial expression of pain or (e) suboptimal hepatic function (ALT > 2 × upper normal limit) or suboptimal renal function (urine output < 1 ml/kg/hour in the 12 hours prior to the study intervention).

The study was approved by the Research Ethics Board of The Ottawa Hospital. Informed consent was obtained from a parent or legal guardian for all enrolled infants by a research assistant or a member of the research team, at arms-length of clinical care.

### Intervention

The study gel was applied to the proposed PICC site by a nurse 'blind' to group assignment (under the supervision of the research assistant), 30 minutes prior to the insertion of the PICC. Tetracaine 4% gel was applied to the skin of each participant allocated to the treatment group; Professional Care Lotion (by Smith-Nephew) was applied to the skin of each participant allocated to the placebo group. An occlusive dressing (sterile Saran Wrap) was applied over the gel. Tubes of 1.5 g of tetracaine were used, of which 1.1 g could be extracted; the dose of gel applied to the skin was therefore 1.1 g. After 30 minutes, the gel was removed and the PICC procedure began.

### Blinding

The study gel was packaged by a single research pharmacist in identical looking ointment jars identified by medication number, which matched the enrolment number. Both gels were white, odorless and had the same consistency. Pre-prepared jars containing tetracaine 4% or placebo gel identified by the medication number were sent to the NICU and stored in the refrigerator. Once a participant was enrolled, the next sequential jar was used for the PICC insertion. The parents, guardians and research team, including outcome assessors, were blind to treatment assignment throughout the study. We did not evaluate the success of our blinding procedure.

### Randomization

Infants were randomized with equal probability (1:1) of being allocated to either placebo or tetracaine 4% gel. The sequence, a random-permuted block with block size of 4, was computer-generated by the study statistician. To help ensure adequate allocation concealment, the computer sequence was kept centrally on computer and could only be activated when there was an eligible baby to randomize. The Research Assistant (RA) completed an eligibility criteria checklist using a personal digital assistant (PDA). Once an affirmative answer was given to all questions the RA synchronized the PDA with a desktop computer. This relayed the PDA answers to the central randomization computer. Upon receipt of an affirmative answer, the central computer released the group assignment for that infant only. For this trial, the group assignment was given as "baby # 1 to receive jar of gel labeled # 1" and so on in sequential order until all babies were allocated. Only the research pharmacist was aware of the randomization code. The randomization sequence was not broken until data analysis was completed.

### Co-interventions

Where possible, both groups received standard currently-practiced non-pharmacological measures of non-nutritive sucking, swaddling and comforting throughout the PICC insertion procedure, as appropriate for gestation.

### Procedure

The PICC (24 Gauge, 1.9 French or 28 Gauge 1.2 French L-Cath Peel Away System; Luther Medical Inc, Tustin, CA) was inserted according to a standardized protocol by a select group of nurses (total of 4) who are certified in PICC insertion. Data were collected during 5 phases of the PICC insertion: (1) Application phase – the PICC nurse examined the skin. She applied the study gel to the appropriate antecubital area and an occlusive dressing (sterile Saran wrap) was applied over the site. After 30 minutes the dressing and the gel were removed. (2) Baseline – 2 The infant remained undisturbed in a position of comfort for 1 minute. (3) Preparation–The site was cleansed according to the PICC protocol. (4) PICC insertion – The central catheter was inserted according to protocol and a dressing was then applied. (5) Recovery – The infant was returned to a position of comfort at which point filming ceased.

A maximum of three attempts were made to insert the PICC. If unsuccessful, the procedure was stopped, a dressing was applied at the puncture site and the infant was returned to his/her previous comfort position. Failed attempts to insert a PICC were recorded as failures and data were analyzed according to treatment allocation.

Physiological indicators of pain (heart rate, blood pressure, respiratory rate, oxygen saturation) were continuously recorded using a Hewlett Packard Neonatal Monitor from the application phase to the recovery phase.

The RA videotaped facial action indicators from baseline to the end of recovery phase with a Sony 8 mm digital Camcorder. The total duration of cry from PICC insertion to recovery was determined from the video camera recordings in non-intubated infants. All data were entered on a personal digital assistant and subsequently uploaded to a central database.

### Outcomes

The primary outcome was the Premature Infant Pain Profile (PIPP) score in the first minute of the PICC insertion phase. The PIPP was specifically developed to assess acute pain in preterm and term infants [13]. It has undergone extensive clinimetric development for procedural pain of heel-stick, venipuncture, intravenous insertion and circumcision. Interrater and intrarater reliability analysis for individual event scores yielded coefficients of 0.93 to 0.96 and 0.94 to 0.98, respectively [13]. One of two trained facial coders from Dr Bonnie Steven's laboratory at the Hospital for Sick Children assessed the video camera recordings of the PICC insertion for three specific validated facial expressions of pain that are part of the PIPP score: brow bulge, nasolabial furrow and eye squeeze. The intrarater and interrater reliability scores of the facial coders were 0.95 and 0.95 (percent agreement) respectively during the study (personal communication, Janet Yamada, February 2005). Coders were blinded to the group allocation of infants. PIPP scores were calculated by one investigator (DH) by combining these behavioral findings with the other components of the PIPP. PIPP scores were assigned every minute during the PICC procedure, from baseline through to the recovery phase. Using the tool as it was originally validated, differences in oxygen saturation and heart rate were compared from baseline to each minute after skin puncture, which marked the beginning of the insertion phase. Each PIPP score was derived by calculating two 30-second intervals for each minute of the procedure. The PIPP score was assigned as the highest value of the two 30 second scores.

The secondary outcome measures were: (i) PIPP scores during the second, third and fourth minute of the insertion phase (compared both independently and longitudinally including the observationduring the first minute of the procedure); (ii) mean heart rate in beats per minute, respiratory rate per minute, blood pressure in mm Hg and 0_2 _saturation in % at the end of baseline, 1 minute into the PICC insertion phase, then 2, 3, 4, 5 and 10 minutes into the insertion phase; (iii) duration of cry, from PICC insertion to recovery; (iv) mean number of attempts required to insert the PICC, success rate and subjective measure of ease/difficulty on a scale of 1 to 5 (1 being very easy and 5 very difficult). Ease of insertion was derived by asking the RN to appraise her assessment of the ease of performing the procedure.

The safety of tetracaine was appraised using the following data: local skin reaction (redness, edema), complete blood count and differential (pre and post intervention), AST and ALT (pre and post intervention), and creatinine levels (post intervention). The biochemical measures were obtained during routine blood sampling in the NICU. All infants' vital signs were monitored throughout and after the intervention and any significant event (apnea/bradycardia, sustained bradycardia or tachycardia, sustained desaturation requiring intervention) was recorded and reported until the recovery phase. All infants were monitored continuously after the procedure and staff was encouraged to contact the research team if there were any concerns.

### Sample size

The research team had discussions with several NICU colleagues who manage infants with pain, focusing on what magnitude of difference in pain scores they would deem clinically important between treatment and placebo group. The consensus was a minimally clinically important difference (MCID) of 3 units on the PIPP score (out of 21 units). The following assumptionswere used: (i) a minimal clinical important difference of 3 units on pain score scale; (ii) an estimated standard deviation of 3.2 units on the pain score scale, based on preliminary results of Ballantyne's trial [12]; (iii) effects of tetracaine may be better or worse than the placebo (two-sided test); (iv) statistical power of 90% and a type I error of 5%. With these assumptions, the expected total sample size necessary to detect that difference in pain score was 48 patients (24 per treatment group). To account for potential technical/equipment problems, 10% was added to the sample size, thus 54 participants were recruited. [14]

One interim analysis was conducted after half of the patients were enrolled. This analysis focused on the primary outcome and safety issues. Using the O'Brien-Fleming criteria for two-sided tests, the alpha-level of this interim analysis was 0.005. The results at that time were inconclusive and the trial continued to the planned recruitment target. In the final analysis, differences were declared statistically significant if the *p *value was 0.048 or less.

### Statistical analysis

The differences in PIPP scores between groups were assessed using an independent Student's t-test. Differences between treatment and control groups in PIPP scores measured during the first 4 minutes of intervention were evaluated using an analysis of variance with repeated measures, adjusting for gender and postnatal age. It has been shown that these variables may influence the level of pain in infants [15].

A Generalized Wilcoxon test was used to assess whether or not the duration of cry was different for the group receiving tetracaine 4% gel and the control group. The difference in number of attempts inserting the PICC was determined using a Poisson regression model. Ease of insertion was compared between study groups with a Mann-Whitney test. Finally, Fisher's exact tests were conducted to assess a difference in success in PICC insertion and frequency of adverse event. Safety comparisons (local skin reaction, anomalies on the CBC, liver or renal function tests) and sequential vital signs for each group were summarized using descriptive statistics.

## Results

**Figure 1:**
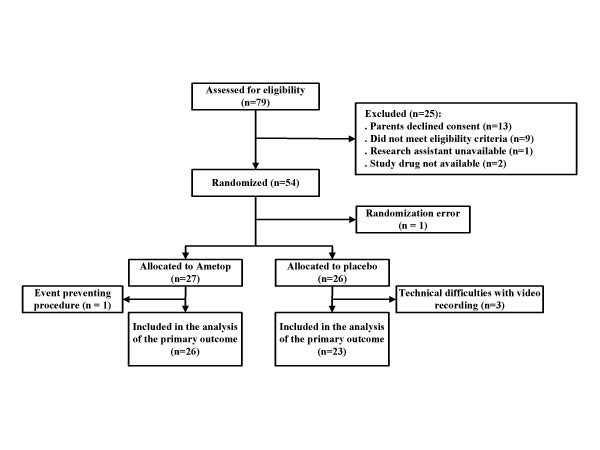
Participant flow through the various stages of the study.

Participants were enrolled in the study between December 2002 and July 2004. Seventy-eight infants were assessed for eligibility during that time, 54 were randomized and primary outcome data were recorded for 49 of these (Figure 1). There was one randomization error and primary outcome was unavailable for 3 infants owing to technical difficulties with the video recordings. In another infant, the PICC had to be postponed owing to persistent bradycardia after the study drug was applied. Excluded patients did not differ from included patients in their baseline characteristics (Table 1).

Baseline characteristics of included infants were similar between groups (Table 3). The mean PIPP score in the first minute was 10.88 in the treatment group as compared to 11.74 in the placebo group (p = 0.53). Although the PIPP scores during the 1^st^, 2^nd^, 3^rd ^and 4^th ^minutes were 0.45 to 1.27 units lower in the tetracaine group as compared to the placebo group, this was not statistically significant (Table 3). A repeated measures ANOVA, adjusted for gender and age in days, revealed no significant differences in PIPP scores between groups over the first 4 minutes (p = 0.330). Median duration of crying in non-intubated infants was 181 (treatment group) versus 68 seconds (placebo group) (p = 0.771). Ease of insertion of the PICC, number of attempts and success rates were similar between groups. Mean heart rate, respiratory rate, mean oxygen saturation and mean blood pressure at the end of baseline, 1, 2, 3, 4, 5 and 10 minutes, were similar between groups (Table 4).

Transient skin erythema was observed in three infants in the tetracaine group and in one infant in the placebo group. One infant in the tetracaine group (25 weeks gestation, 491 g, just over 48 h old) had a sustained bradycardia (heart rate of 80/min) but stable saturation and blood pressure after insertion of a nasogastric tube while the study gel was on his skin. The PICC was postponed and the infant recovered after 45 minutes. No other adverse events were noted throughout the study. No significant changes were noted in any patient's CBC, ALT or creatinine in either group (Table 5).

**Figure 2:**
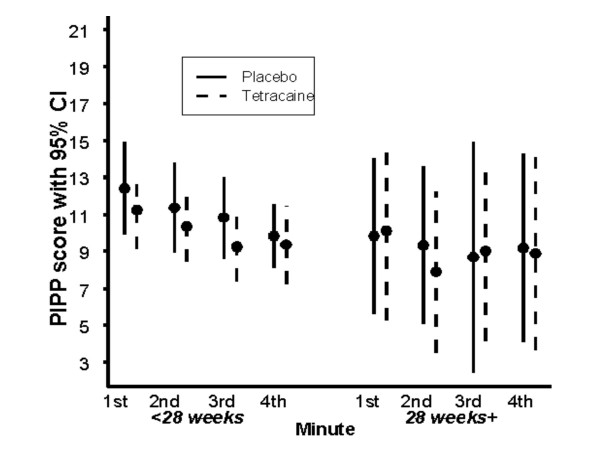
PIPP scores according to gestational age.

Primary clinical validation of the PIPP suggests smaller infants could have a lower PIPP score [13]. Since a significant proportion of our population was less than 28 weeks gestation (n = 35), we conducted a post hoc analysis of the primary outcome, stratified for gestational age (i.e. less than 28 weeks or 28 weeks or more). No difference was found in the sequential PIPP scores, either in the treatment or in the control groups, on the basis of this gestation cut-off (Figure 2). PIPP scores were slightly higher in more immature infants by up to 1–2 points.

## Discussion

In this study, 1.1 g of topical tetracaine 4%, applied to premature infants 30 minutes before insertion of a PICC, did not significantly reduce procedural pain as measured by the PIPP tool. We had sufficient statistical power to detect at least a 3-unit difference in PIPP scores between the treatment groups, but less than a third (0.86 units) of this difference was observed. Despite the statistical result we believe our findings are relevant for clinicians at the bedside, when they deliberate how best to alleviate pain during insertion of a PICC in infants.

Two previous trials have evaluated topical anesthetics before a PICC insertion in infants. In a randomized trial, Garcia et al. [16] compared EMLA to placebo for percutaneous insertions of central venous lines in infants less than 1500 g. They found that infants in the treatment group had a smaller increase in their heart rates during the procedure. No formal pain tool was used and the sample was small (n = 13). More recently, Ballantyne et al. [12] evaluated the benefit of 1.0 g of tetracaine in infants 27 to 41 weeks gestation undergoing a PICC insertion. As in our study, no significant difference in the PIPP scores could be seen over the first 3 minutes of the procedure. The PIPP tool was used and the technique to apply a tourniquet (a sterile rubber catheter) was similar to the one used in this study. Infants in that study had a mean gestational age of 33 weeks and their PIPP scores overall were 2–23 points lower than ours.

There are several explanations for the lack of significant difference between the treatment and the placebo groups. Like other trials exploring ways to minimize pain in infants, this study was subject to potential problems related to the use of assessment processes and tools. Manually coding the videotapes and pain assessment is probably always open to human error. We conducted random calibration exercises to re-verify the PIPP scores and tested inter-rater and intra-rater reliability during the study; however, some margin of error may have remained.

To our knowledge, we included the smallest and most immature infants ever enrolled in a randomized controlled trial involving tetracaine. More than half of our population (n = 35) was less than 28 weeks gestation. These infants are representative of those requiring a PICC in tertiary care centers. However, data are limited on multidimensional pain responses and their assessment in infants less than 28 weeks gestation. Previous validation data by Stevens et al. showed more immature infants to have lower PIPP scores than more mature ones [13]. Only 9 infants less than 28 weeks gestation were included in that study. Our post hoc analysis of the primary outcome, stratified for gestational age, demonstrated higher PIPP scores in infants less than 28 weeks (up to 1 to 2 points) than in those 28 weeks or greater. This may partly explain the difference in PIPP scores between our study and Ballantyne's. The PIPP tool continues to undergo clinimetric testing and is probably going to keep evolving with more research and use in very preterm infants.

The recommended pediatric dose of tetracaine was used. The drug was removed from its original package to preserve blinding. There is no specific recommendation from the manufacturer on how long the drug is active once removed from the package; however, crystallization occurs with evaporation of the water-based gel leading to drug inactivity and should be felt upon application. This was not observed in our study, but we cannot rule out that some of the doses of tetracaine may have become inactive. Biologically, tetracaine blocks the action potential and we are not aware of a specific reason why it would fail to do so, even in immature infants whose skin technically could absorb more medication rather than less. Recent findings in pharmacogenomics and pharmacogenetics suggest that individual genetic factors may explain inter-individual differences in response to medications [17]. We cannot exclude the possibility that this could be a confounding variable in the extremely premature population, although randomization should have limited the impact.

Given the immaturity of enrolled infants and the lack of safety data in this population, we chose to apply the gel for 30 minutes only, as did Ballantyne. The recommended duration of application for venous cannulation is 45 minutes. There is evidence from pediatric literature that an increased duration of application leads to better skin anesthesia [18]. Jain et al. evaluated time to skin anesthesia in infants 27 to 42 weeks gestation and found that 64% had local skin anesthesia after 30 minutes and 72% after 45 minutes [7]. Applying the gel for 45 minutes may have improved efficacy and this is a limitation of the study.

Although tetracaine has been shown effective in children to prevent the pain associated with venipuncture or venous cannulation [19-23], subcutaneous vaccination [24] or Port-a-Cath puncture [25], it has failed to be beneficial for heel pricks in newborn infants [10,11]. A plausible reason is that, for heel pricks, the squeezing of the heel rather than the skin puncture is the most painful event. The pain from squeezing would not be affected by a topical anesthetic. Inserting a PICC requires more than a simple skin puncture. Infants are restrained, a tourniquet is applied and the procedure can last up to 30 minutes. It has been demonstrated in previous studies that handling and immobilization lead to behavioral and physiological reactivity [26,27]. Thus, the skin puncture is unlikely to be the only painful trigger during a PICC procedure

Our choice of primary outcome (difference of 3 on the PIPP score) was not based on a pilot study. It appeared reasonable and clinically meaningful to aim for a difference of 3. However, Ballantyne et al. [12] could not find a difference of 2 and sucrose appears to decrease PIPP scores by about 2 in venipunctures [28]. Tetracaine may simply not reduce pain in a clinically meaningful way in the very preterm population undergoing a PICC.

Another possible limitation of the study is that we did not record the number of painful procedures infants had undergone before randomization. This has been shown to influence the intensity of the reaction to pain [1,29]. Both groups had similar postnatal age and gestational age; however, the number of painful procedures may have differed between infants, though this should have been balanced by the randomization.

PIPP scores for infants in our study were in the "moderate pain" range. As topical anesthetics have not yet been shown effective, we would advocate that centers inserting PICCs consider adding a systemic analgesic to their current procedural pain relief guideline. Opioids have potential side effects, such as cardiovascular instability and urinary retention as well as possibly leading to worse neurodevelopmental outcomes [30], which limit their use on a regular basis. However, for limited use such as for procedural pain relief, the advantages may outweigh the risks [31].

No infant enrolled received oral sucrose, as they were too immature and not on adequate feeding volumes to meet the eligibility criteria for sucrose according to our policy. Sucrose has been shown effective in reducing pain related to heel pricks and venipunctures in infants 25 to 41 weeks gestation up to 28 days of age [29]. Furthermore, an RCT demonstrated that sucrose compared favorably with EMLA in term newborns undergoing a venipuncture [32]. We speculate that sucrose may have helped modulate the pain, at least in the first few minutes. Some centers in Europe use sucrose more liberally in extremely immature infants and report no adverse effect (personal communication, R. Carbajal, May 2005). Sucrose may be an adjunct or alternative to consider in infants undergoing a PICC insertion.

## Conclusion

Tetracaine 4%, when applied for 30 minutes, was not beneficial in decreasing procedural pain associated with a PICC in very small infants. A longer application time of tetracaine should be considered in future trials. Future trials should also consider including sucrose into their intervention to decrease pain associated with insertion of a PICC, even in very immature infants who are not feeding.

## Competing interests

The author(s) declare that they have no competing interests.

## Authors' contributions

BL designed the study, received peer reviewed funding to conduct the trial and drafted the manuscript. RS participated in the design of the study and contributed to the drafting of the manuscript. DH contributed to the study design, enrolled patients, collected data and had significant input into the drafting of the manuscript. IG participated in the design of the study, performed the statistical analysis and contributed to drafting the manuscript. CB participated in the design of the study. DM participated in the design of the study and in the drafting of the manuscript. All authors read and approved the final manuscript.

## Pre-publication history

The pre-publication history for this paper can be accessed here:



## Figures and Tables

**Table 1:** Baseline characteristics of excluded and included participants

**Characteristics**	**Excluded (n = 16)**	**Included (n = 53)**	**Difference (95%CI)**	**p-value**
Birth weight in grams, mean (sd)	983.1 (242.38)	915.2 (294.29)	67.96 (-93.45, 229.37)	0.406
Completed weeks of gestation, mean (sd)	27.7 (1.99)	27.1 (2.23)	0.66 (-0.58, 1.90)	0.330
Male, n (%)	11 (68.8)	23 (43.4)	25.4 (-2.4, 46.5)	0.093

**Table 2:** Baseline characteristics

**Characteristics**	**Placebo (n = 26)**	**Tetracaine (n = 27)**
Birth weight in grams, mean (sd)	950.6 (294.94)	881.1 (295.14)
Completed weeks of gestation, mean (sd)	26.9 (2.04)	27.2 (2.43)
Male, n (%)	11 (57.7)	12 (44.4)
Age in days, mean (sd)	6.6 (3.01)	6.8 (3.25)

**Table 3:** Study outcomes

**Outcome**	**n**	**Placebo**	**n**	**Tetracaine**	**Difference (95%CI)**	**p-value**
PIPP, 1^st ^minute, mean (sd)	23	11.74 (4.73)	26	10.88 (4.70)	0.86 (-1.86, 3.58)	0.530
PIPP, 2^nd ^minute, mean (sd)	23	10.83 (4.59)	25	9.56 (4.34)	1.27 (-1.33, 3.87)	0.331
PIPP, 3^rd ^minute, mean (sd)	22	10.23 (4.65)	25	9.16 (4.32)	1.07 (-1.57, 3.71)	0.419
PIPP, 4^th ^minute, mean (sd)	23	9.65 (3.74)	25	9.20 (4.78)	0.45 (-2.06, 2.96)	0.718
Duration of cry in seconds, median (range)	8	68 (0, 477)	7	181 (0, 944)	-78 (-539, 117)	0.771
Ease of insertion, median (range)	26	2 (1, 5)	26	2 (1, 5)	0 (-1, 0)	0.613
Number of attempts, median (range)	26	2 (1, 3)	26	1 (1, 4)	0 (0, 1)	1.000
PICC successfully inserted, n (%)	26	24 (93.3)	26	19 (73.1)	19.2 (-1.9, 39.2)	0.140

**Table 4:** Physiological parameters during the insertion phase

**Outcome**	**n**	**Placebo**	**n**	**Tetracaine**
BP at 1 minute, mean (sd)	16	48.7 (9.3)	18	44.5 (5.0)
BP at 2 minutes, mean (sd)	17	49.6 (9.9)	20	46.4 (7.7)
BP at 3 minutes, mean (sd)	13	47.4 (9.4)	19	43.1 (6.1)
BP at 4 minutes, mean (sd)	17	45.8 (6.5)	17	43.2 (5.2)
BP at 5 minutes, mean (sd)	15	43.6 (5.4)	19	45.6 (12.9)
BP at 10 minutes, mean (sd)	15	46.3 (7.6)	16	43.0 (6.8)
				
Heart rate at 1 minute, mean (sd)	26	162.6 (12.5)	26	162.4 (11.7)
Heart rate at 2 minutes, mean (sd)	26	159.8 (17.0)	26	161.3 (9.1)
Heart rate at 3 minutes, mean (sd)	26	159.3 (15.2)	26	157.5 (10.7)
Heart rate at 4 minutes, mean (sd)	26	156.9 (14.1)	26	157.3 (14.5)
Heart rate at 5 minutes, mean (sd)	26	156.4 (13.7)	26	155.2 (10.2)
Heart rate at 10 minutes, mean (sd)	21	149.2 (15.6)	19	155.4 (10.2)
				
O2 sat at 1 minute, mean (sd)	25	90.9 (4.9)	26	92.6 (4.2)
O2 sat at 2 minutes, mean (sd)	26	91.4 (4.9)	26	92.8 (3.9)
O2 sat at 3 minutes, mean (sd)	25	91.3 (5.3)	26	93.2 (3.9)
O2 sat at 4 minutes, mean (sd)	26	91.6 (5.4)	26	92.8 (4.8)
O2 sat at 5 minutes, mean (sd)	25	92.0 (5.4)	25	92.6 (4.4)
O2 sat at 10 minutes, mean (sd)	21	91.7 (8.2)	19	94.0(4.2)
				
Respiration at 1 minute, mean (sd)	26	55.7 (15.9)	26	51.3 (13.9)
Respiration at 2 minutes, mean (sd)	26	53.9 (13.1)	26	51.8 (13.0)
Respiration at 3 minutes, mean (sd)	26	58.5 (17.5)	26	50.6 (12.7)
Respiration at 4 minutes, mean (sd)	26	50.5 (14.5)	26	55.8 (17.6)
Respiration at 5 minutes, mean (sd)	26	54.3 (16.8)	26	52.1 (14.3)
Respiration at 10 minutes, mean (sd)	20	55.3 (15.6)	17	50.7 (11.5)

**Table 5:** Safety data

**Outcome**	**n**	**Placebo**	**n**	**Tetracaine**
Change from baseline in ALT (U/l), median (range)	21	0.0 (-9.0, 20.0)	21	1.0 (-6.0, 10.0)
Change from baseline in creatinine (μmol/l), median (range)	23	-7.0 (-101.0, 23.0)	23	-6.0 (-30.0, 27.0)
Change from baseline in neutrophil count (x10^9 ^/l), median (range)	15	-0.7 (-4.3, 12.3)	13	0.6 (-4.7, 7.6)
Change from baseline in platelet count (x10^9 ^/l), median (range)	22	42.0 (-278.0, 260.0)	23	7.0 (-118.0, 202.0)
